# The role of SLC34A2 in intestinal phosphate absorption and phosphate homeostasis

**DOI:** 10.1007/s00424-018-2221-1

**Published:** 2018-10-20

**Authors:** Joanne Marks

**Affiliations:** Department of Neuroscience, Physiology and Pharmacology, Royal Free Campus, University College London, Rowland Hill Street, London, NW3 2PF UK

**Keywords:** NaPi-IIb, Phosphate, Paracellular transport, Intestine, Homeostasis

## Abstract

There has recently been significant interest in the concept of directly targeting intestinal phosphate transport to control hyperphosphatemia in patients with chronic kidney disease. However, we do not have a complete understanding of the cellular mechanisms that govern dietary phosphate absorption. Studies in the 1970s documented both active and passive pathways for intestinal phosphate absorption. However, following the cloning of the intestinal SLC34 cotransporter, NaPi-IIb, much of the research focused on the role of this protein in active transcellular phosphate absorption and the factors involved in its regulation. Generation of a conditional NaPi-IIb knockout mouse has demonstrated that this protein is critical for the maintenance of skeletal integrity during periods of phosphate restriction and that under normal physiological conditions, the passive sodium-independent pathway is likely be the more dominant pathway for intestinal phosphate absorption. The review aims to summarise the most recent developments in our understanding of the role of the intestine in phosphate homeostasis, including the acute and chronic renal adaptations that occur in response to dietary phosphate intake. Evidence regarding the overall contribution of the transcellular and paracellular pathways for phosphate absorption will be discussed, together with the clinical benefit of inhibiting these pathways for the treatment of hyperphosphatemia in chronic kidney disease.

## Introduction

The regulation of phosphate homeostasis is achieved via complex interactions between the kidney, intestine and bone, and several endocrine factors [7]. It is widely accepted that the kidneys play the major role in maintaining extracellular phosphate concentrations within their narrow limits. Consequently, the majority of the research focus with regard to understanding the cellular mechanisms and regulation of phosphate transport has concentrated on the processes that occur in the proximal tubule.

Historically, less is known about the cellular pathways that govern intestinal phosphate absorption. However, with the desire to develop novel drugs to diminish dietary phosphate absorption in patients with chronic kidney disease (CKD), there has been renewed interest in understanding the processes involved in intestinal phosphate handling. In particular, the generation of a conditional knockout mouse for the intestinal SLC34 cotransporter, NaPi-IIb, has revealed some significant and unexpected insights into the role of this protein in intestinal phosphate absorption and phosphate homeostasis. This review aims to present evidence that NaPi-IIb is critical for intestinal phosphate absorption during periods of phosphate restriction or during ontogenesis, but that during adulthood under normal physiological conditions, the sodium-independent pathway would play a more dominant role.

## Intestinal phosphate absorption

Studies in the 1970s, using the triple-lumen perfusion technique in healthy volunteers, reported that the jejunum had a higher phosphate transport rate than the ileum and that in the jejunum, transport could be resolved into sodium-dependent and sodium-independent components [30, 65]. This regional profile for phosphate absorption was comparable to the observations described in rats in the same decade and led to the conclusion that intestinal phosphate absorption occurred predominately in the proximal small intestine [64]. In 2005, this assumption was questioned when it was shown that in mice, phosphate absorption occurs maximally in the ileum [51]. At this time, studies demonstrated that the profile for phosphate absorption in each rodent species broadly relates to the regional expression of NaPi-IIb [21, 44, 51]. Taken together with the knowledge that the major regulators of intestinal phosphate absorption (dietary phosphate and 1,25-dihydroxyvitamin D_3_) induce alterations in protein levels of NaPi-IIb [23, 51], it was widely accepted to be the main sodium-dependent intestinal phosphate transporter, and it played a significant role in intestinal phosphate absorption [18, 21, 54].

Over the past 10 years, the role of NaPi-IIb in intestinal phosphate absorption and phosphate homeostasis has been intensely investigated. While it is still considered to be the major phosphate transporter present at the enterocyte brush border membrane (BBM), likely accounting for 90% of transcellular sodium-dependent transport [54], there are emerging reports documenting the presence of SLC20 proteins at this cellular location [3, 11, 21]. In rats, PiT1 and more recently PiT2 proteins have been reported to be present in intestinal regions with a high absorptive capacity for phosphate [11, 21]. In mice, PiT2 has been detected in the ileum, but the regional profile has not been established [52]. There are conflicting reports as to whether PiT1 is regulated by dietary phosphate intake. Giral et al. were unable to demonstrate any change in protein levels after 7 days of a low-phosphate diet, while Candeal et al. showed a small but significant increase in abundance in the duodenum, but not jejunum, after 5 days, with a more robust change occurring in both segments after 10 days. The reason for this discrepancy is unclear given that both studies used male rats (Sprague Dawley vs. Wistar, respectively) of comparable age that were fed diets with the same phosphate content. The most likely explanation for the differences is that long-term phosphate restriction is required for substantial changes in the levels of this protein to occur [12, 21]. Levels of PiT2 protein also appear to adapt to changes in dietary phosphate content, but these changes occur more rapidly than PiT1 and mirror the time course for changes in NaPi-IIb [12]. While these studies demonstrate that SLC20 levels are regulated by dietary phosphate intake, proof for a functional role of these proteins in intestinal phosphate absorption is lacking. Controversially, although Candeal and colleagues documented changes in both SLC34 and SLC20 proteins in response to altered dietary phosphate, they propose that an additional sodium-dependent transport system plays a major role in intestinal phosphate absorption under certain conditions [12]. However, the exact identity of this system and physiological relevance remains unknown.

The overall contribution of the sodium-dependent versus sodium-independent components of intestinal phosphate absorption remains a constant debate. In 1977, Walling demonstrated that there was a linear relationship between phosphate absorption and intraluminal phosphate concentrations at values above 1 mmol/L [64]. Similar findings were reported by others at this time [65, 67] and were interpreted that phosphate absorption was probably an active process at values below 1 mmol/L and that passive absorption would occur at higher luminal concentrations. Of significance was the statement by Walton and Grey that ‘Our results are entirely consistent with this hypothesis but not sufficient to prove it’ [65]. Thirty years on, we are now only just amassing sufficient evidence to substantiate this hypothesis. Importantly, the cloning of NaPi-IIb in 1998 [26] led researchers to focus on this transporter, and combined with the use of experimental approaches and phosphate concentrations to favour this transport pathway, it was concluded that this was the dominant route for intestinal phosphate absorption [18, 21, 54]. However, we now know that phosphate concentrations present in the bulk phase of the intestinal contents are in the millimolar range [16, 29, 35, 48]. Recent results from studies utilising these concentrations to evaluate phosphate transport in rats [48], NaPi-IIb knockout mice [29] and Caco-2 cells [11] have promoted the consensus that sodium-independent transport is indeed likely to be dominant under normal physiological conditions.

## Cloning and tissue expression of NaPi-IIb

A full-length cDNA of SLC34A2 was first identified in 1998 from a mouse embryo EST clone and was shown to have a high homology (73%) to mouse NaPi-IIa, particularly in the regions that represent transmembrane segments [26]. The gene was detected in mouse small intestine, colon, lung, kidney and testis [26], and in humans, it was subsequently detected at high levels in lung, salivary glands, mammary glands and small intestine [49]. It is now known that constitutive deletion of the SLC34A2 gene in mice is embryonically lethal [58].

The cloned sequence codes for a 697 amino acid protein, with a molecular weight of 108 kDa for the glycosylated form commonly detected in adult mouse small intestine [26]. Partially glycosylated NaPi-IIb (~ 88 kDa) has been reported in 14-day-old mouse intestine, with the suggestion that full glycosylation occurs at the point of transition between suckling and weaning [4]. As previously discussed, the profile for NaPi-IIb expression in the small intestine shows significant species differences, but regardless of the intestinal region, the protein is localised to the enterocyte BBM along the entire length of the villus [21, 23, 26]. Interestingly, using autoradiography, it has been shown that phosphate uptake, at least at luminal phosphate concentrations that favour NaPi-IIb-mediated transport, is restricted to the mid-upper regions of the villi [45]. The difference between NaPi-IIb protein expression pattern and its function is probably a consequence of the time required for full functional expression of the protein during enterocyte migration and maturation along the villus and the development of the electrochemical gradient required for phosphate uptake.

NaPi-IIb is localised in tissues other than the small intestine. In the lung, the protein is restricted to the apical membrane of alveolar type II cells and is thought to be required for surfactant production [56, 63]. Mutations in SLC34A2 are associated with pulmonary alveolar microlithiasis [55], and recently, dysregulation of NaPi-IIb has been implicated in tumorigenesis and progression of non-small cell lung cancer [66]. In human salivary glands, NaPi-IIb is found in both the parotid and submandibular glands, where it has been localised to the basolateral membrane of acini and the apical membrane of duct cells and is proposed to be involved in phosphate secretion and reabsorption, respectively [27]. Although not usually associated with renal phosphate handling, the protein has been reported to be localised to the basolateral membrane of the collecting duct and/or the ascending limb of the loop of Henle, and levels are upregulated in response to a high-phosphate diet [60]. The proposed function of NaPi-IIb in these tubular segments is to aid phosphate excretion during high dietary phosphate intake; this however requires clarification [60].

## Functional characterisation of NaPi-IIb in intestinal phosphate absorption

Characterisation of NaPi-IIb in *Xenopus* oocytes revealed this protein to be a high-affinity sodium-dependent phosphate cotransporter with an apparent *K*_m_ for phosphate of ~ 50 μmol/L and a *K*_m_ for sodium of ~ 30 mmol/L [26]. It has been shown to be exclusively sodium-dependent, to preferentially transport divalent phosphate and to be electrogenic translocating one net positive charge per transport cycle due to the 3:1 Na^+^:Pi stoichiometry [19]. Given the observation that phosphate uptake using intestinal BBM vesicles has similar characteristics, with an affinity for phosphate of ~ 0.1 mmol/L [6, 43], it was concluded that NaPi-IIb was the protein responsible for the sodium-dependent component of intestinal phosphate absorption.

**Fig. 1:**
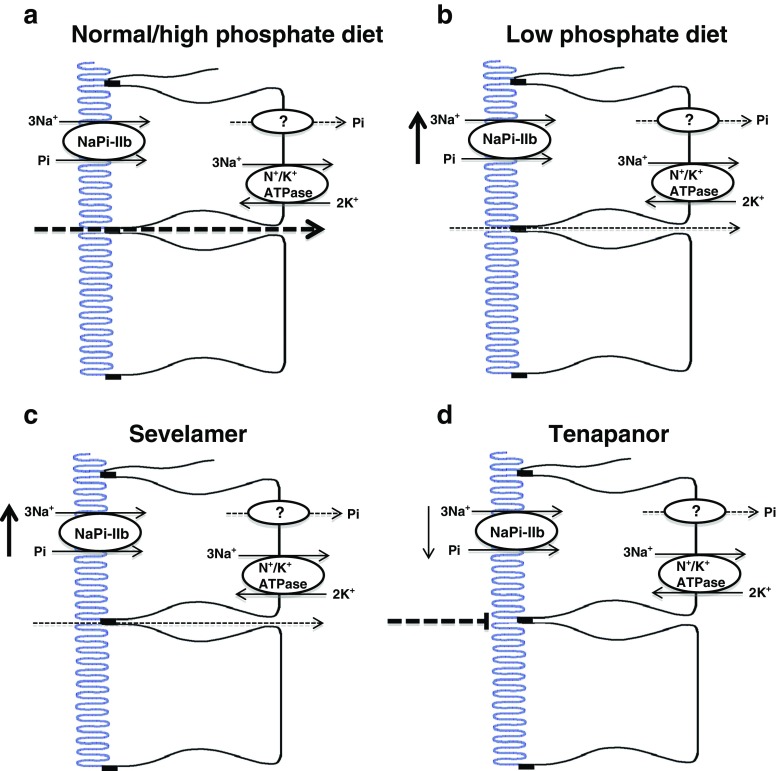
Potential adaptations in intestinal phosphate absorption in response to changes in dietary phosphate intake, or treatment with phosphate binders or the NHE3 inhibitor, tenapanor. **a** Ingestion of a normal or high-phosphate diet provides a luminal phosphate gradient that favours the paracellular pathway, with NaPi-IIb likely functioning at its maximal rate. Renal adaption occurs in response to the high dietary phosphate intake in order to maintain phosphate homeostasis [25, 51]. **b** In contrast, during ingestion of a low-phosphate diet, the luminal phosphate gradient is reduced and the contribution of the paracellular pathway diminishes. NaPi-IIb protein levels increase and this pathway becomes the dominant route for phosphate absorption [51]. Studies from NaPi-IIb^−/−^ mice suggest that the adaptation in NaPi-IIb is required to protect bone during phosphate restriction [36]. **c** Phosphate binders reduce the luminal phosphate concentration and therefore the gradient for paracellular phosphate absorption. Recent studies in NaPi-IIb^−/−^ mice have shown a potential compensatory upregulation in NaPi-IIb following sevelamer treatment [54]. **d** Tenapanor has been proposed to reduce paracellular phosphate absorption via changes in trans-epithelial electrical resistance. Interestingly, the inhibitor also induces a downregulation in NaPi-IIb protein levels as a means of preventing compensation by this pathway [34]. The basolateral pathway responsible for phosphate efflux from the enterocyte is unknown and whether it adapts during these conditions requires investigation

Many researchers, myself included, subsequently used 0.1 mmol/L phosphate to favour NaPi-IIb-mediated transport when investigating the mechanisms involved in the regulation of intestinal phosphate absorption. These studies revealed that a low-phosphate diet and 1,25-dihydroxyvitamin D_3_ are the major regulators in intestinal phosphate absorption [23, 31]. In response to chronic dietary phosphate restriction, upregulation of mRNA and protein levels of NaPi-IIb occur in the jejunum [21] and ileum [14, 51] of rats and mice, respectively, and these alterations correlate with functional changes in absorption [21, 51]. Based on these findings, it is clear that active transcellular phosphate absorption adapts in response to reduced oral phosphate intake and that it is likely that this pathway is dominant under these conditions (Fig. 1b). In contrast, when luminal phosphate concentrations increase following ingestion of a phosphate-rich diet, NaPi-IIb will be functioning at its maximal rate and the luminal phosphate gradient will instead favour the paracellular pathway (Fig. 1a). In this context, dietary phosphate restriction would not only increase NaPi-IIb-mediated transport but also lower the gradient for passive transport across the epithelium, prompting a rapid switch from the dominance of one pathway to the other [36]. Importantly, the idea that NaPi-IIb-mediated transport is only dominant during dietary restriction is not new. In 1977, Walling commented that their unpublished observations in adult rats maintained on an adequate phosphate intake showed no active absorption of phosphate by any gut segment [64].

## Intestinal phosphate absorption in NaPi-IIb knockout mice

The idea that NaPi-IIb may not be the dominant pathway under normal dietary phosphate conditions is also supported by recent studies using mice with intestinal ablation of NaPi-IIb. When fed a standard diet (0.6–0.8% phosphate), NaPi-IIb^−/−^ mice have only moderate faecal phosphate wasting [25, 54, 57]. In addition, NaPi-IIb^+/−^ mice fed on a 0.8% phosphate diet show no change in faecal phosphate excretion, even though NaPi-IIb mRNA and protein levels and sodium-dependent phosphate uptake measured using BBM vesicles are decreased by more than half [25]. In line with the suggestion that NaPi-IIb is functionally more dominant during dietary phosphate restriction, maintenance of NaPi-IIb^−/−^ mice on a low (0.1%)-phosphate diet results in an overall 40–45% increase in faecal phosphate excretion relative to wild-type animals [36], while, importantly, NaPi-IIb^−/−^ mice maintained on a high (0.9–1.2%)-phosphate diet show either no significant increase [29], or a decrease [36], in faecal phosphate excretion compared to wild-type mice on the same diet, demonstrating the larger contribution of passive transport after consumption of a high-phosphate load.

Studies utilising changes in post-prandial serum phosphate levels to access the acute impact of NaPi-IIb ablation on intestinal phosphate absorption also suggest a relatively small role of NaPi-IIb. In experiments designed to maximise the potential contribution of the protein, Sabbagh et al. maintained NaPi-IIb^−/−^ mice on a low (0.02%)-phosphate diet for 1 week prior to exposure to an acute phosphate load. In these experiments, there was only a 50% reduction in post-prandial phosphate absorption compared to wild-type animals [54]. The authors acknowledge that their estimates of NaPi-IIb-mediated transport likely represent the maximal potential contribution and that under normal dietary phosphate intake, the overall percentage of active NaPi-IIb-mediated transport to post-prandial phosphate absorption is likely to be lower. Similar findings are also evident from in vitro transport studies using different phosphate concentrations in the uptake buffer to compare the overall contribution of sodium-dependent and sodium-independent phosphate transport across the intestinal epithelium. From these studies, it is clear that intestinal ablation of NaPi-IIb in mice abolishes sodium-dependent phosphate uptake into ileal BBM vesicles by 95% [25] and in ileal everted sac’s by 90% [54], when using 0.1 mmol/L and 1.2 mmol/L phosphate, respectively. However, recent studies, also using the everted sac technique, but evaluating phosphate absorption at 4 mmol/L, suggest that there is no significant difference in sodium-dependent or sodium-independent phosphate transport between wild-type and knockout animals, in contrast to transport measured at 1.2 mmol/L when sodium-dependent transport was significantly reduced [29].

## Is sodium-independent intestinal phosphate absorption transcellular or paracellular?

It is still unclear whether the sodium-independent pathway for phosphate absorption occurs via the paracellular or transcellular route. Early studies demonstrated that the pathway is measurable not only in vivo but also when using BBM vesicles. At this time, the sodium-independent pathway was shown not to be regulated by 1,25-dihydroxyvitamin D_3_ or chronic dietary phosphate adaptation [31, 39]. However, increased sodium-independent phosphate transport has recently been reported using BBM vesicles prepared from rats acutely switched from a low- to high-phosphate diet [11]. In addition, in the human Caco2BBE intestinal cell line, phosphate transport has been proposed to be solely sodium-independent, that it is promoted by high levels of phosphate in the growth medium, a response which is mainly caused by changes in *V*_max_ of the transport process and depends on de novo RNA and protein synthesis [11]. Taken together, these results suggest that there may be a transcellular component to sodium-independent phosphate absorption.

An alternative, and perhaps a more accepted consensus, is that sodium-independent phosphate transport occurs via the paracellular route. Claudins are integral to tight junction function, permitting or preventing paracellular transport through their sealing or pore-forming characteristics [2, 22]. A number of the pore-forming claudins have cation or anion selectivity or are permeable to water [22], with evidence linking claudin-2, claudin-12 and claudin-15 to paracellular calcium absorption in the small intestine (reviewed in [1]). Whether specific tight junction proteins are involved in modulating paracellular phosphate absorption is currently not known. However, recent studies using tenapanor provided strong evidence for a link between tight junction function and phosphate permeability. This small-molecule inhibitor of the sodium/hydrogen exchanger (NHE3) acts locally in the gastrointestinal tract to inhibit sodium absorption, but also significantly reduces intestinal phosphate absorption in healthy volunteers [34] and improves hyperphosphatemia in both rodents and humans with CKD [8, 37]. It has been suggested to induce conformational changes in the tight junctions, as a result of intracellular proton retention caused by NHE3 inhibition, leading to increased trans-epithelial electrical resistance (TEER) and thus a reduction in permeability to phosphate. Importantly, tenapanor also decreases the expression of NaPi-IIb to prevent compensation by the transcellular route [34] (Fig. 1d).

While King et al. propose that conformational changes within the tight junction complexes, rather than changes in the levels or localisation of specific claudins, affect paracellular phosphate permeability in response to tenapanor [34], it is interesting to note that alterations in mRNA and protein levels of both pore-forming and sealing claudins occur in the proximal and distal small intestine of NaPi-IIb^−/−^ mice [29]. Ikuta et al. speculate that these changes might influence sodium-independent phosphate transport but do not definitively associate them with adaptation in transport per se. It is however plausible that alteration in tight junction composition occurs to compensate for the reduced transcellular phosphate absorption. Such a phenomenon has been reported in mice lacking calbindin D9k, the intestinal calcium-binding protein critical for transcellular calcium absorption, where overexpression of claudins 2 and 12 has been proposed to compensate for the insufficient transcellular absorption [28].

## NaPi-IIb and the gut-renal axis

In 2007, Berndt et al. provided evidence for an active role for the intestine in the maintenance of phosphate homeostasis. They proposed that a gut-derived factor was released in response to ingestion of dietary phosphate and that this factor rapidly modulated renal phosphate reabsorption to prevent large post-prandial fluctuations in serum phosphate levels [5]. The so called ‘intestinal phosphatonin’ was suggested to act independently of changes in plasma phosphate concentration or parathyroid hormone (PTH) [5]. The finding that matrix extracellular phosphoglycoprotein (MEPE), a protein typically secreted from bone [53] that has a phosphaturic effect on the kidney [59], is present in the intestinal epithelium, specifically the duodenum [3, 47], led to the suggestion that this may be the factor proposed by Berndt et al. [47]. However, subsequent studies were unable to confirm the existence of this intestinal phosphatonin, but instead found that acute renal adaptation to an oral phosphate load requires PTH [40, 62]. With regard to MEPE, it is interesting to note that a recent study has demonstrated that uremic rats maintained on a high-phosphate diet have significantly increased levels of intestinal MEPE expression. Since MEPE inhibits jejunal phosphate absorption [46], it is possible that instead of acting as an endocrine hormone, it may directly inhibit dietary phosphate absorption in an autocrine or paracrine fashion, thus preventing acute changes in post-prandial phosphate levels following ingestion of a high-phosphate load.

Other evidence in support of the existence of a gut-renal axis comes from experiments using NaPi-IIb^−/−^ mice. These demonstrate that chronic impairment of intestinal phosphate transport activates compensatory renal reabsorption in order to maintain phosphate homeostasis [25, 29, 50, 54]. The first publication documenting this effect used male mice maintained on a 0.6% phosphate diet and concluded that the reduction in urinary phosphate excretion was a consequence of increased NaPi-IIa protein levels brought about by a decrease in the levels of the osteocyte-derived phosphaturic hormone, FGF-23 [54]. Subsequent studies have confirmed that NaPi-IIb ablation prompts compensatory reductions in urinary phosphate excretion but have provided conflicting results regarding the mechanism responsible. In adult male NaPi-IIb^−/−^ mice maintained on a 0.8% or 0.9% phosphate diet, there is no change in intact FGF-23 levels [25, 50], NaPi-IIa protein expression [25, 29, 50] or sodium-dependent transport activity [25, 29], even though the mice have the expected decrease in urinary phosphate excretion. In contrast, adult female and 4-week-old male NaPi-IIb^−/−^ mice maintained on a 0.8% or 0.9% phosphate diet have a reduction in circulating FGF-23 and elevated NaPi-IIa protein levels [25, 50]. The reason for the difference in response to gender, age and levels of dietary phosphate is not known. It is possible that there are different thresholds to trigger the degree of compensatory renal change depending on the long-term phosphate status and that when phosphate demand is highest, this induces the FGF-23/NaPi-IIa response to maximally enhance phosphate reabsorption with the aim of preserving phosphate balance. Alternatively, or perhaps as part of the above mechanism, it has been suggested that the more dominant renal effect seen in females than males maybe due to differences in the proteolytic processing of FGF-23 [25].

Finally, in context of the sodium-independent pathway, NaPi-IIb^−/−^ mice fed a high 1.2% phosphate diet have significantly elevated phosphaturia in comparison to those maintained on a 0.8% phosphate diet [36]. Although this is slightly lower compared to wild-type mice on the same diet, it does again demonstrate that the non-saturable route for phosphate absorption is dominant during phosphate loading. As expected, the phosphaturia in both wild-type and knockout mice is associated with elevated FGF-23 and PTH levels and a reduction in 1,25 dihydroxyvitamin D_3_, with no significant difference between the two groups [36]. Therefore, although it has been suggested that NaPi-IIb could act as a sensing molecule regulating the release of the unknown gut-derived phosphaturic factor, it is more plausible based on the above observations that both acute and chronic gut-renal interactions are dependent on endocrine factors balancing renal phosphate reabsorption to match intestinal phosphate absorption.

## Is NaPi-IIb a suitable target to control hyperphosphatemia in chronic kidney disease?

Current strategies to control hyperphosphatemia in CKD patients include dietary phosphate restriction and the use of phosphate binders. These approaches are often associated with poor patient compliance due to the high-phosphate content of the typical Western diet, and the frequent dosing and thus large pill burden, and often significant gastrointestinal intolerance, caused by phosphate binders [13, 15]. In addition, given the likely dominant role of NaPi-IIb when intestinal phosphate concentrations are low, these strategies may even favour dietary phosphate absorption. Indeed, mice with adenine-induced CKD that are treated with the phosphate binder sevelamer have elevated NaPi-IIb levels (Fig. 1c). This adaptation was used to explain why binder treatment was less effective in wild-type mice compared to uremic NaPi-IIb^−/−^ mice, where treatment completely attenuated the hyperphosphatemia associated with renal insufficiency [57].

The observation that NaPi-IIb ablation in uremic mice causes a sustained decrease, although not always complete normalisation, of serum phosphate levels [50, 57] was used to support the idea that NaPi-IIb is a suitable treatment target for preventing or reducing hyperphosphatemia in CKD patients. Furthermore, niacin and its derivative nicotinamide, proposed inhibitors of the type II phosphate transporter family [32, 68], also have a positive impact on hyperphosphatemia in rodents [17] and humans with CKD and ESRD (reviewed in [20, 41]), although their use is associated with some adverse effects that potentially preclude their clinical use [24, 42]. These observations led to the development of ASP3325, a specific small-molecule NaPi-IIb inhibitor, which in several rat models was shown to alleviate CKD-induced hyperphosphatemia [61]. Unfortunately, subsequent clinical studies failed to provide evidence that ASP3325 influences urinary phosphate or faecal phosphate excretion in normal healthy individuals, or that it reduces serum phosphate levels in those with ESRD [38]. This lack of efficacy was suggested to be a result of either a small or negligible role of NaPi-IIb in humans [38]. However, the puzzle remains as to why niacin and nicotinamide effectively lower serum phosphate levels in CKD and ESRD patients if NaPi-IIb does not play a major role in phosphate absorption in humans. Larsson et al. suggested that it may be that these compounds offer a superior target engagement compared to ASP3325, or that their phosphate-lowering capacity may be caused by other pleiotropic effects [38]. An alternative explanation and mode of action for nicotinamide may be similar to that reported for tenapanor [34], that it acts on tight junctions to reduce paracellular phosphate absorption and induces a decrease in NaPi-IIb expression to prevent compensation by the transcellular pathway.

## Role of NaPi-IIb in bone development and skeletal integrity

The skeleton acts as a large reservoir for phosphate, containing approximately 80% of total body phosphate; this is primarily found complexed with calcium in the form of hydroxyapatite crystals [9]. In healthy adults, bone formation is in balance with bone resorption; however, during infancy and adolescence, the skeletal phosphate requirement is elevated to allow for rapid bone development and growth. In this context, it is well documented that in rats, intestinal phosphate absorption is highest during suckling and weaning and that the age-related decline in absorption correlates with decreased gene and protein levels of NaPi-IIb [4, 10, 69]. A critical role for NaPi-IIb during ontogenesis is also supported by studies in NaPi-IIb^+/−^ mice. At 4 weeks of age, these mice have impaired intestinal phosphate transport and hypophosphatemia, while in contrast, at 20 weeks of age when the skeletal demand for phosphate is not as high, the compensatory reduction in urinary phosphate excretion seen in both age groups of NaPi-IIb^+/−^ mice is sufficient to maintain normal serum phosphate concentrations [50].

It should, however, be noted that NaPi-IIb^+/−^ mice show comparable weight gain and growth as their wild-type littermates, suggesting that either the residual NaPi-IIb present in the intestine is sufficient to provide enough phosphate for normal skeletal development, or that another pathway for absorption also contributes to the phosphate supply. In keeping with the latter suggestion, early studies by Borowitz and Ghishan investigating the impact of age on intestinal phosphate transport documented higher rates of sodium-independent as well as sodium-dependent phosphate transports in younger animals [10]. Therefore, although current evidence highlights the importance of NaPi-IIb in intestinal phosphate transport during ontogenesis, the role of the sodium-independent pathway perhaps deserves reinvestigation given that the high-phosphate content of the milk consumed during suckling and weaning would generate a favourable gradient for passive transport across the intestinal epithelium.

In addition to the role for NaPi-IIb in intestinal phosphate absorption during growth and development, the protein also appears to be involved in maintaining bone integrity. Recent studies by Knopfel et al. have shown that NaPi-IIb^−/−^ and wild-type mice maintained for 2 weeks on a low-phosphate diet have normal serum phosphate and almost no urinary phosphate excretion. However, in NaPi-IIb^−/−^ mice, there is exaggerated calcium excretion, lower bone mineral density and a higher number of osteoclasts, leading to the suggestion that bone resorption may be enhanced in NaPi-IIb^−/−^ mice releasing both calcium and phosphate at the cost of bone mass loss [36]. Therefore, NaPi-IIb appears to be important in protecting bone, particularly during long-term phosphate restriction.

Bone disease and extra-skeletal calcification are considered to be key characteristics of CKD-mineral bone disorder [33]. Studies investigating the role for NaPi-IIb in bone health during CKD have shown that treatment of uremic NaPi-IIb^−/−^ mice with sevelamer provides significant phosphate control and that this is associated with improvements in bone histomorphometric parameters [57]. Importantly, these effects were not as pronounced in sevelamer-treated wild-type mice, or untreated knockout mice, perhaps reflecting roles for both the sodium-dependent and sodium-independent phosphate absorption pathways in the management of bone disease in CKD.

## Summary

Following the cloning of NaPi-IIb 20 years ago, its molecular characteristics and regulation were investigated in detail. However, more recently, knowledge of the phosphate concentrations present in the intestinal lumen, the generation of a conditional NaPi-IIb knockout mouse and the development of novel inhibitors of phosphate transport have provided us with a more comprehensive understanding of the role of NaPi-IIb in intestinal phosphate absorption and phosphate homeostasis. There is a growing consensus that at dietary phosphate levels seen in the typical Western diet, NaPi-IIb would be saturated and paracellular transport would be the dominant route for phosphate absorption. In contrast, consumption of a diet low in phosphate would not only increase NaPi-IIb protein levels but also lower the gradient for passive transport across the epithelium, prompting a switch from the dominance of one pathway to the other. The importance of NaPi-IIb in phosphate homeostasis is however highlighted by the finding that it protects bone during dietary phosphate restriction.

There are also intriguing new observations that claudin proteins are altered following NaPi-IIb ablation. It is possible that changes in the levels of these tight junction proteins may evoke compensatory modifications to paracellular phosphate absorption in response to reduced transcellular transport. In this context, inhibition of the paracellular pathway with the NHE3 inhibitor, tenapanor, also causes NaPi-IIb downregulation, presumably to prevent compensatory absorption by this pathway. Whether there are common cellular mechanisms regulating these two potentially interlinked pathways remains to be seen.
